# Supplementary material for: Efficient and thermally stable organic solar cells based on small molecule donor and polymer acceptor
Source: Nat Commun. 2019 Jul 22;10:3271. doi: 10.1038/s41467-019-10984-6 (PMC6646397; doi:10.1038/s41467-019-10984-6)
Supplement: Supplementary file 1 — Supplementary Information [file 41467_2019_10984_MOESM1_ESM.pdf]

Supplementary Information

Efficient and thermally stable organic solar cells based on small molecule donor and polymer acceptor

Zhang *et al.*

Supplementary Figures

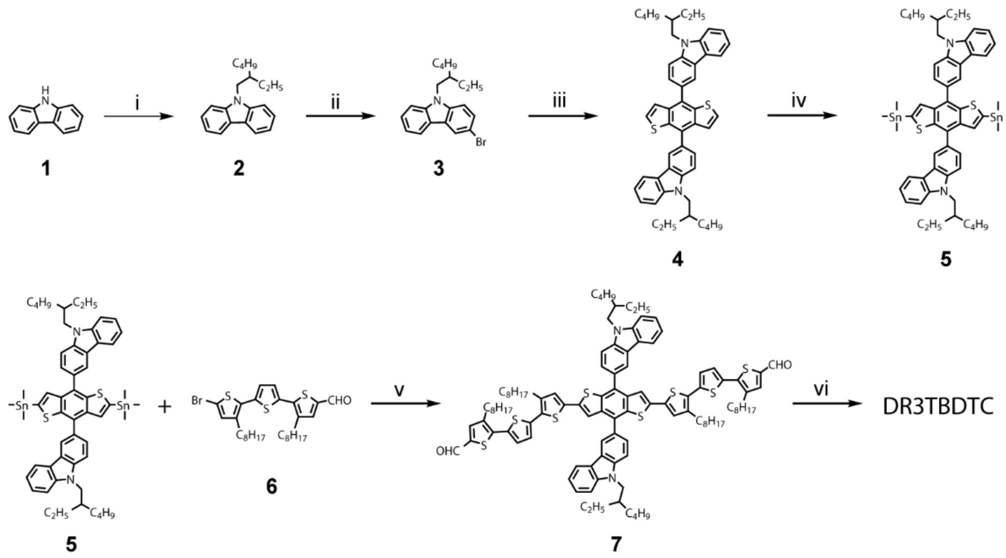

Supplementary Figure 1 Synthetic route of DR3TBDTC. Reagents and conditions: (i) 3-(bromomethyl)heptane, sodium hydride, *N*,*N*-dimethylformamide, r.t.; (ii) *N*-bromosuccinimide, chloroform, 0 ^{o}C to 60 ^{o}C; (iii) *n*-butyllithium, tetrahydrofuran, –78 ^{o}C; benzo[1,2-*b*:4,5-*b'*]dithiophene-4,8-dione, 50 ^{o}C; stannous chloride, dilute hydrochloric acid (10%), r.t.; (iv) *n*-butyllithium, tetrahydrofuran, –78 ^{o}C to r.t.; chlorotrimethylstannane, –78 ^{o}C to r.t.; (v) tetrakis(triphenylphosphine)palladium, toluene, 110 ^{o}C; (vi) 3-ethylrhodanine, chloroform, piperidine, 65 ^{o}C.

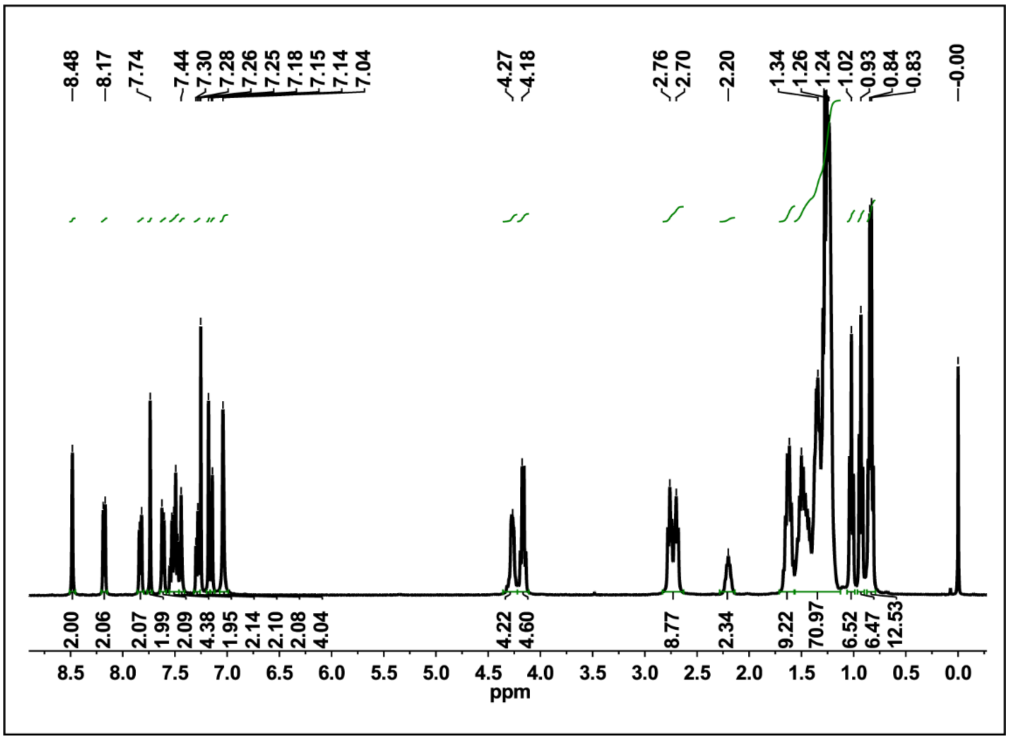

Supplementary Figure 2 ^{1}H NMR of DR3TBDTC.

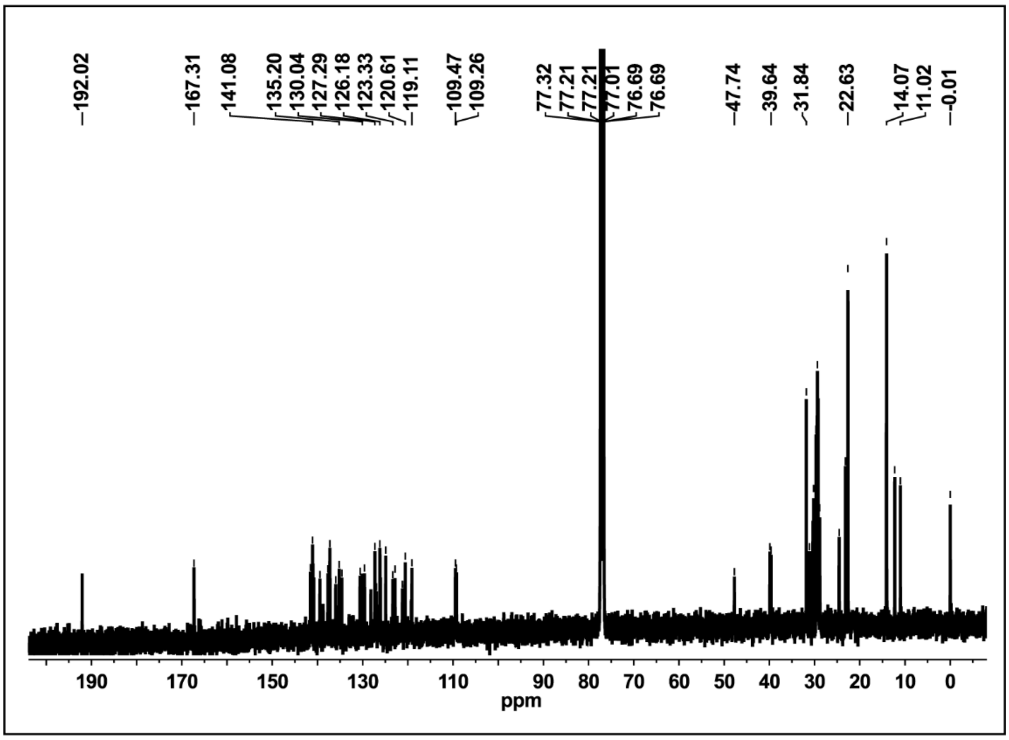

Supplementary Figure 3 ^{13}C NMR of DR3TBDTC.

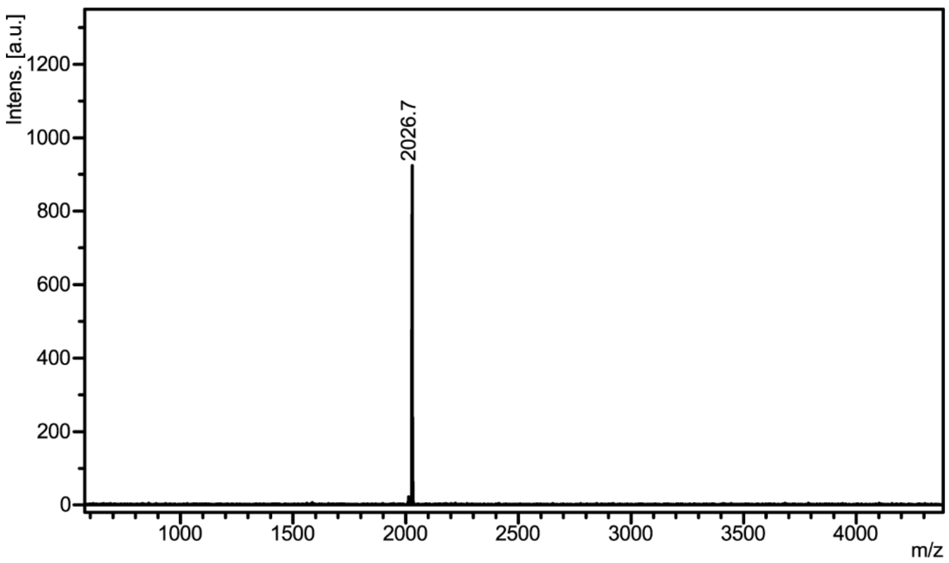

Supplementary Figure 4 The whole MS (MALDI-TOF) plot of DR3TBDTC.

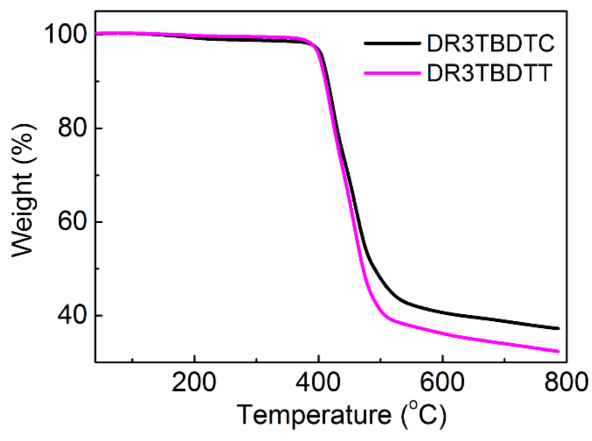

Supplementary Figure 5 TGA plot of DR3TBDTC and DR3TBDTT with a heating rate of 10 °C min^{-1} under N_2 atmosphere.

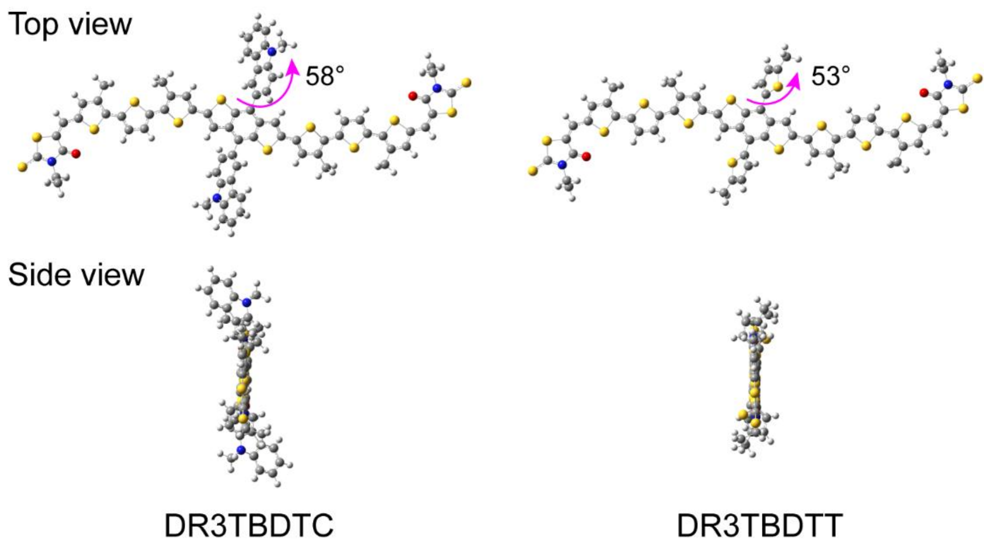

Supplementary Figure 6 The top view and side view of the optimized geometries for DR3TBDTC and DR3TBDTT calculated by DFT/B3LYP/6-31G*.

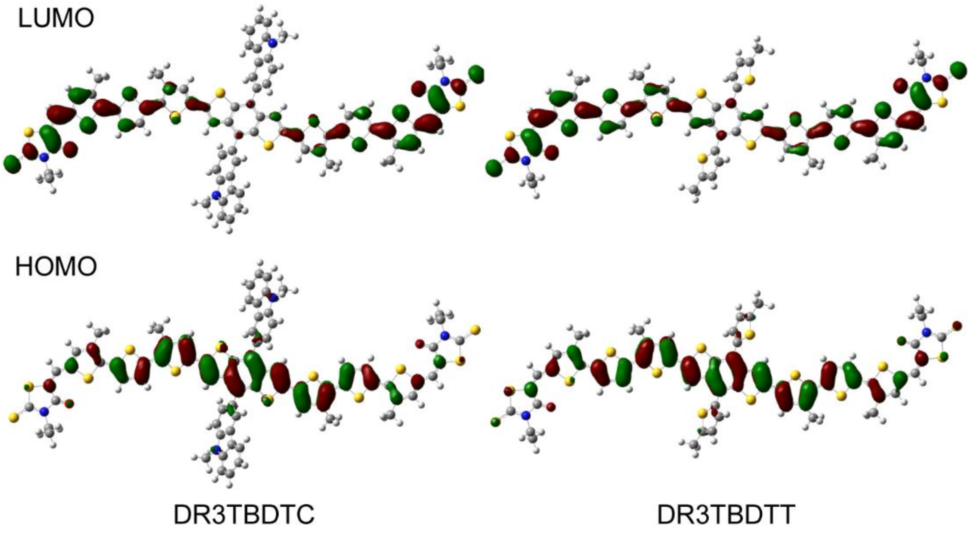

Supplementary Figure 7 The optimized frontier molecular orbitals for DR3TBDTC and DR3TBDTT calculated by DFT/B3LYP/6-31G*.

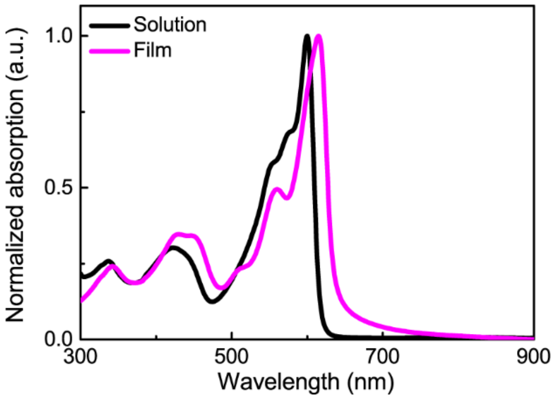

Supplementary Figure 8 Absorption spectra of PBN-11 in solution and in film.

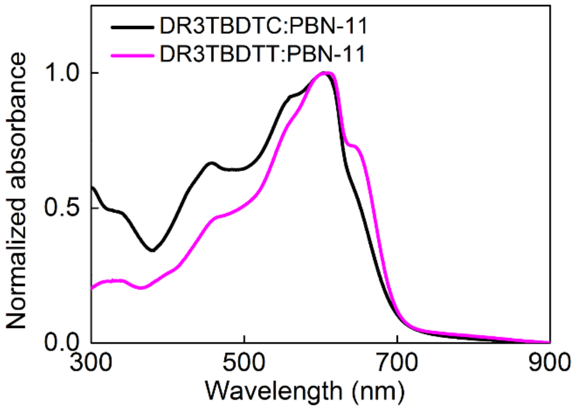

Supplementary Figure 9 Absorption spectra of DR3TBDTC:PBN-11 and DR3TBDTT:PBN-11 blend films.

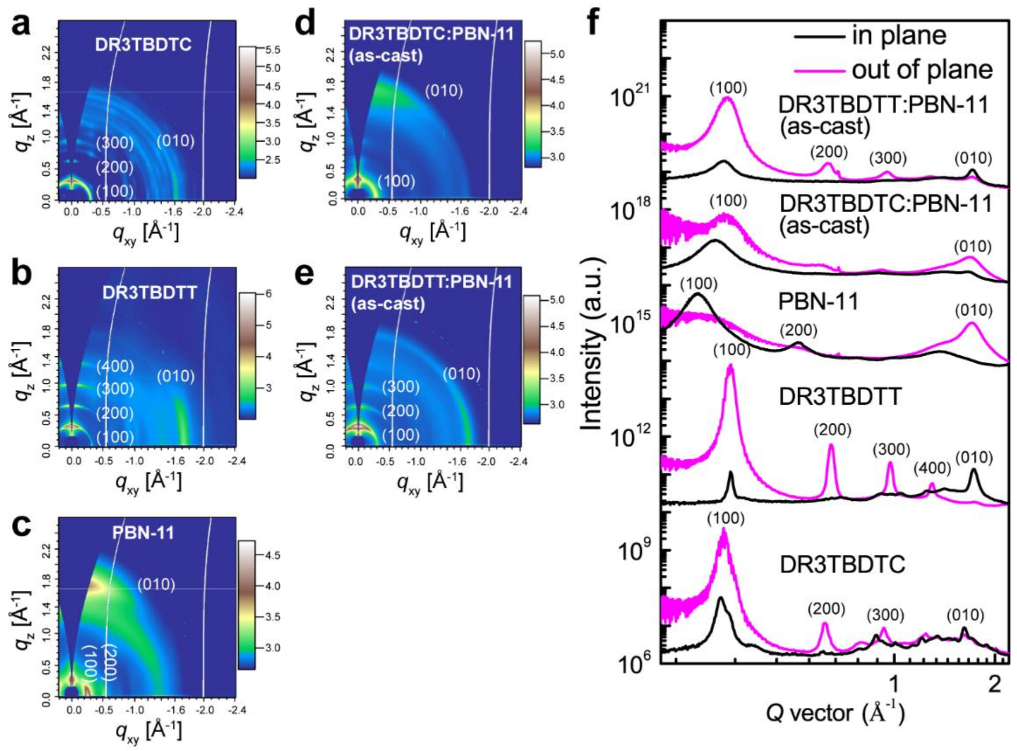

Supplementary Figure 10 2D-GIWAXS patterns of the pure (a) DR3TBDTC, (b) DR3TBDTT, (c) PBN-11 films, the as-cast (d) DR3TBDTC:PBN-11 and (e) DR3TBDTT:PBN-11 blend films, (f) 1D Linecuts of the corresponding 2D-GIWXAS patterns in the in-plane and out-of-plane directions.

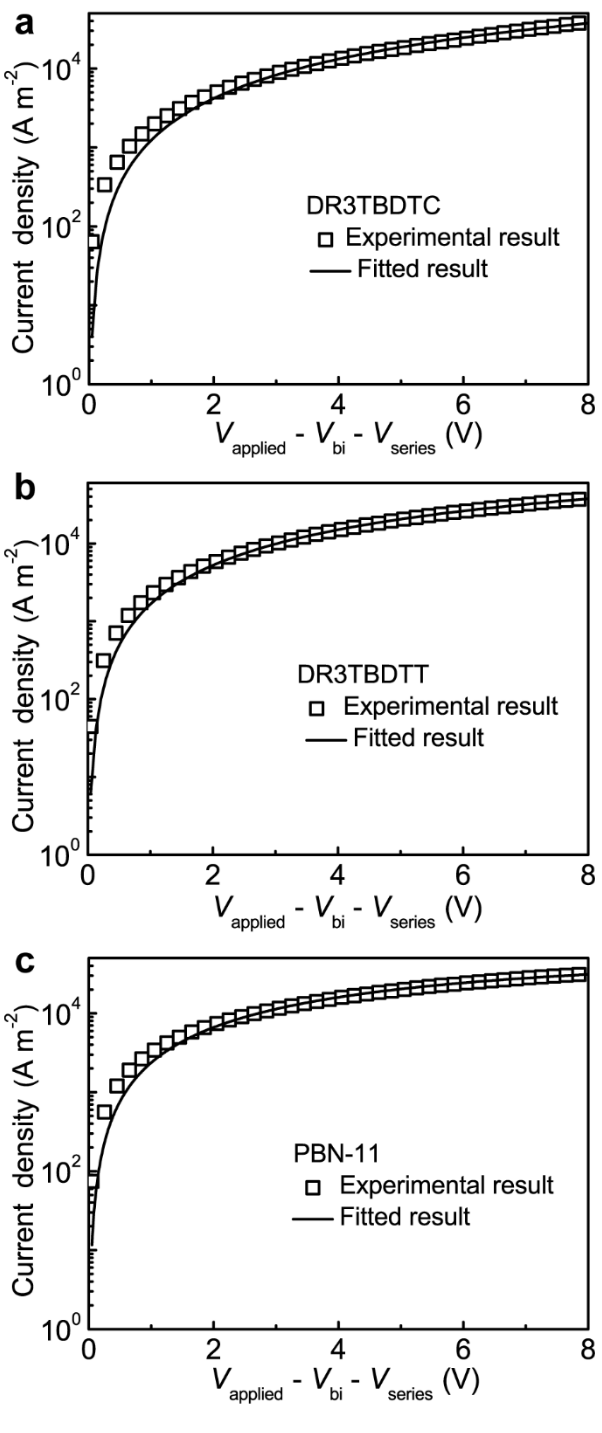

Supplementary Figure 11 Space-charge-limited $J-V$ plots for the hole-only devices of (a) pure DR3TBDTC film, (b) pure DR3TBDTT film, and (c) the electron-only device of the pure PBN-11 film.

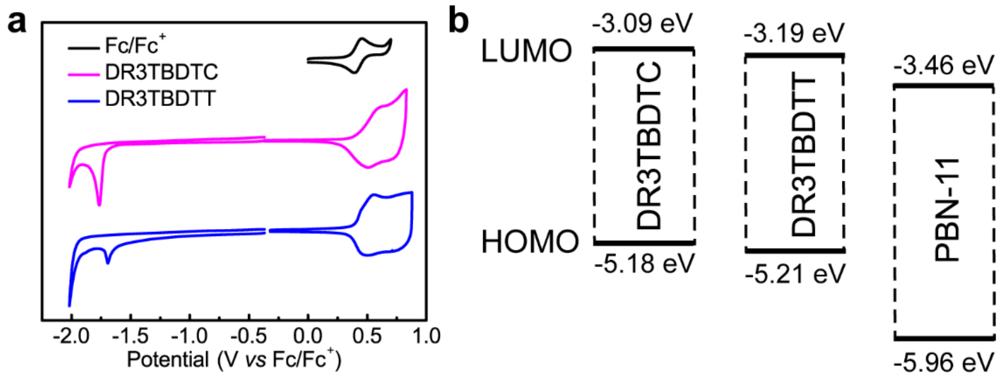

Supplementary Figure 12 a Cyclic voltammetry of DR3TBDTC and DR3TBDTT. The ferrocene/ferrocenium (Fc/Fc^{+}) couple was provided for an internal reference. **b** The energy level diagrams of DR3TBDTC, DR3TBDTT and PBN-11.

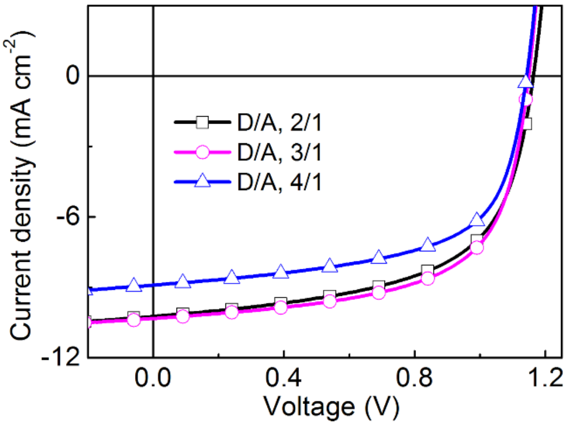

Supplementary Figure 13 J−V plots of the OSCs based on DR3TBDTC:PBN-11 blend films with different weight ratios.

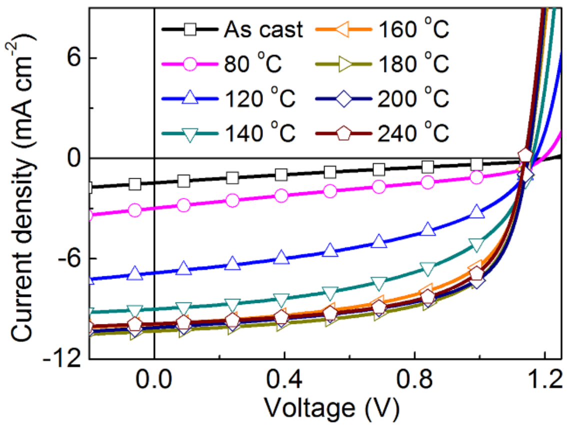

Supplementary Figure 14 $J{-}V$ plots of the OSCs based on DR3TBDTC:PBN-11 blend (3:1, w/w) films annealed at different temperature.

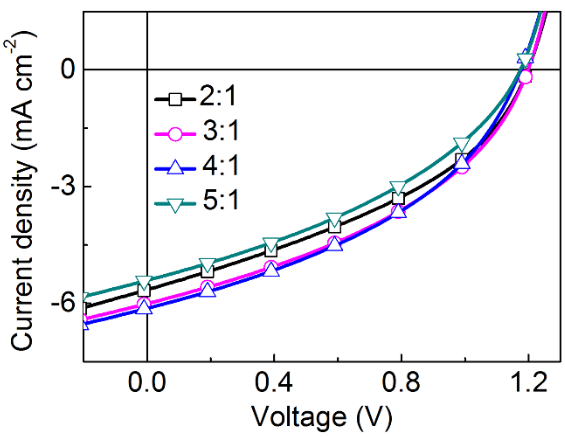

Supplementary Figure 15 *J*–*V* plots of the OSCs based on DR3TBDTT:PBN-11 blend films with different weight ratios.

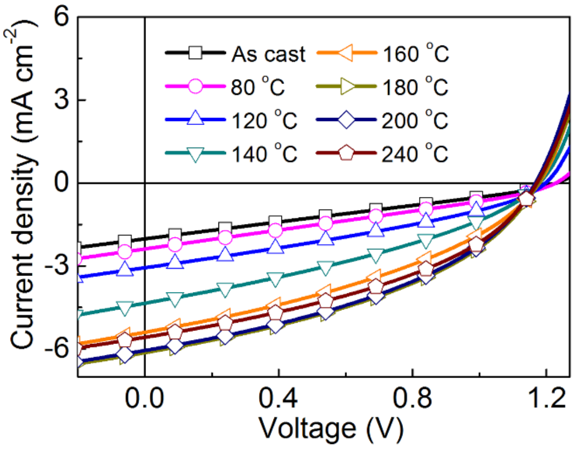

Supplementary Figure 16 *J*−*V* plots of the OSCs based on DR3TBDTT:PBN-11 blend (4:1, w/w) films annealed at different temperature.

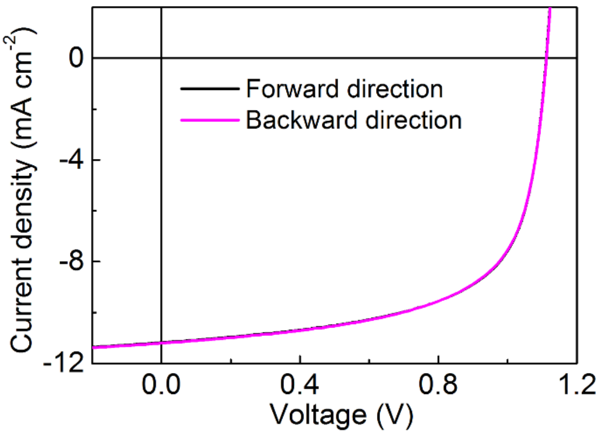

Supplementary Figure 17 J–V plots of the OSCs based on DR3TBDTC:PBN-11 blend (3:1, w/w) films annealed at 180 °C measured in both forward and backward scan directions.

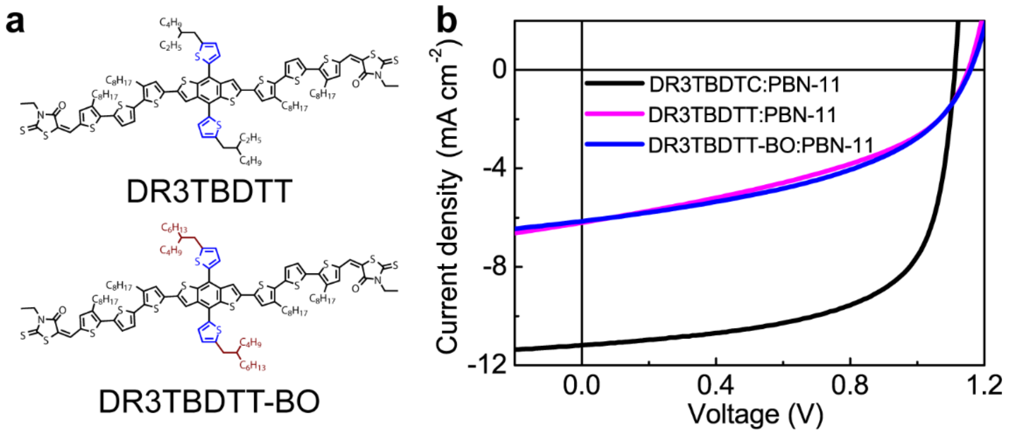

Supplementary Figure 18 The chemical structure of DR3TBDTT and DR3TBDTT-BO, and the *J–V* plots of the OSC devices based on DR3TBDTC:PBN-11, DR3TBDTT:PBN-11 and DR3TBDTT-BO:PBN-11 blends under the illumination of AM1.5G, 100 mW cm^{-2}.

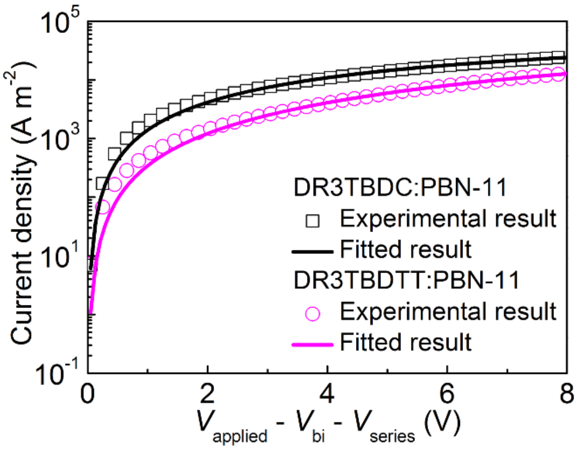

Supplementary Figure 19 Space-charge-limited $J-V$ plots for hole-holy devices of DR3TBDTC:PBN-11 and DR3TBDTT:PBN-11 blend films.

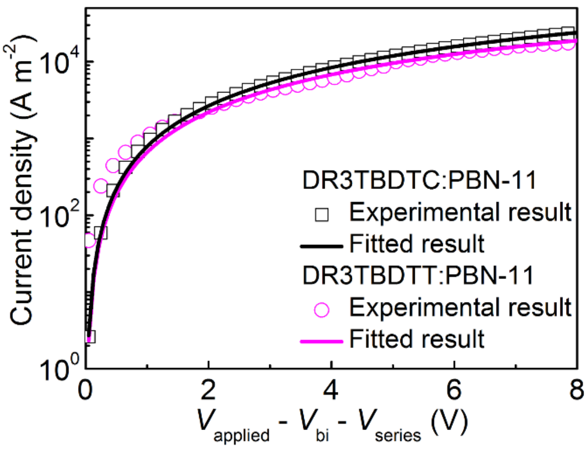

Supplementary Figure 20 Space-charge-limited $J-V$ plots for electron-only devices of DR3TBDTC:PBN-11 and DR3TBDTT:PBN-11 blend films.

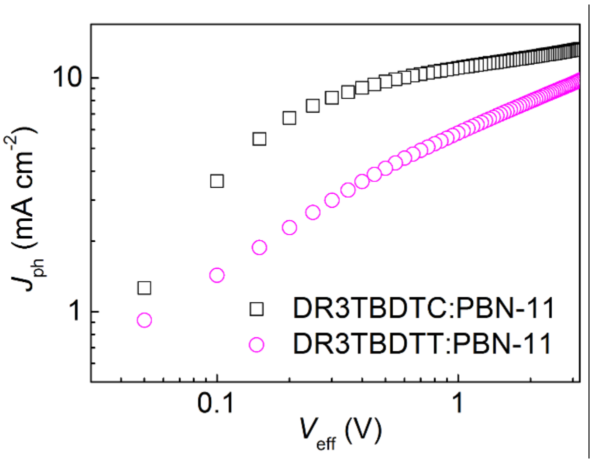

Supplementary Figure 21 J_{ph} versus V_{eff} plots of the OSC devices based on DR3TBDTC:PBN-11 and DR3TBDTT:PBN-11 blend films.

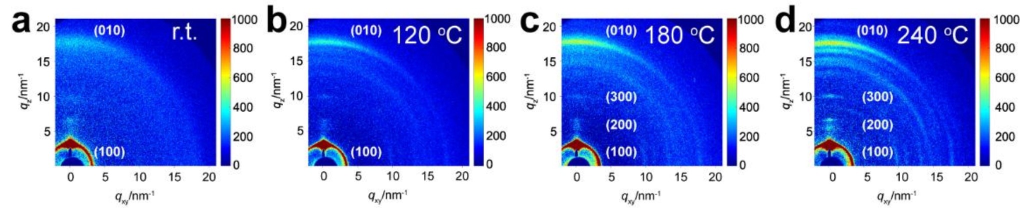

Supplementary Figure 22 GIWAXS patterns of DR3TBDTC:PBN-11 blends annealed at different temperature for 20 minutes: (a) r.t., (b) 120 ºC, (c) 180 ºC, (d) 240 ºC. The reflection signals become stronger with the increasing annealing temperature, indicating increased crystallinity of DR3TBDTC.

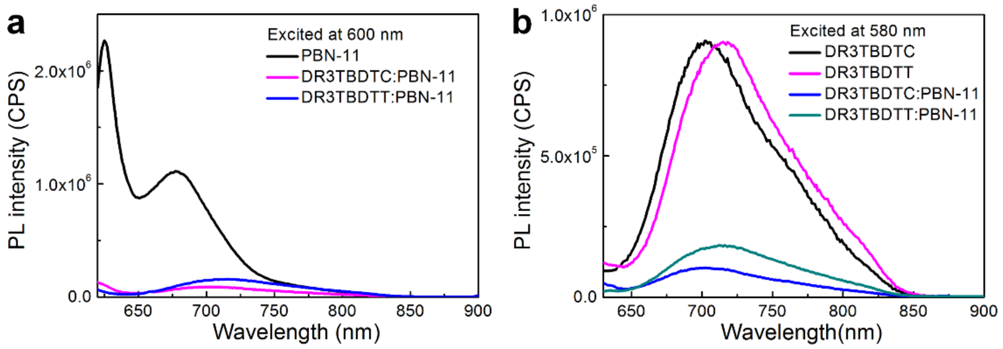

Supplementary Figure 23 PL spectra of (a) pure PBN-11 film, DR3TBDTC:PBN-11 and DR3TBDTT:PBN-11 blend films (excited at 600 nm), (b) pure DR3TBDTC film, pure DR3TBDTT film, DR3TBDTC:PBN-11 and DR3TBDTT:PBN-11 blend films (excited at 580 nm).

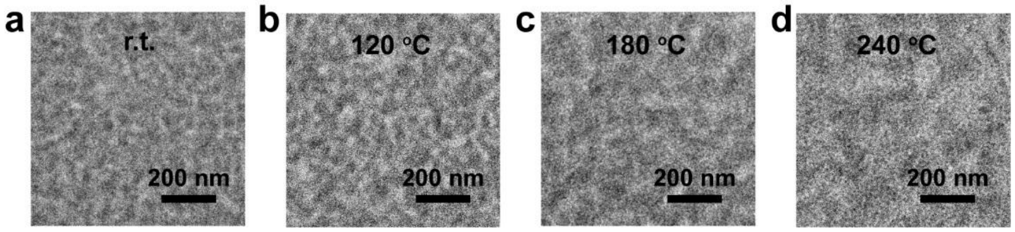

Supplementary Figure 24 TEM images of DR3TBDTC:PBN-11 blends annealed at different temperature for 20 minutes: (a) r.t., (b) 120 ºC, (c) 180 ºC, (d) 240 ºC. The white and dark regions become larger and their contrast becomes obvious as the annealing temperature increases, indicating the relatively large and pure domains are formed in the blend.

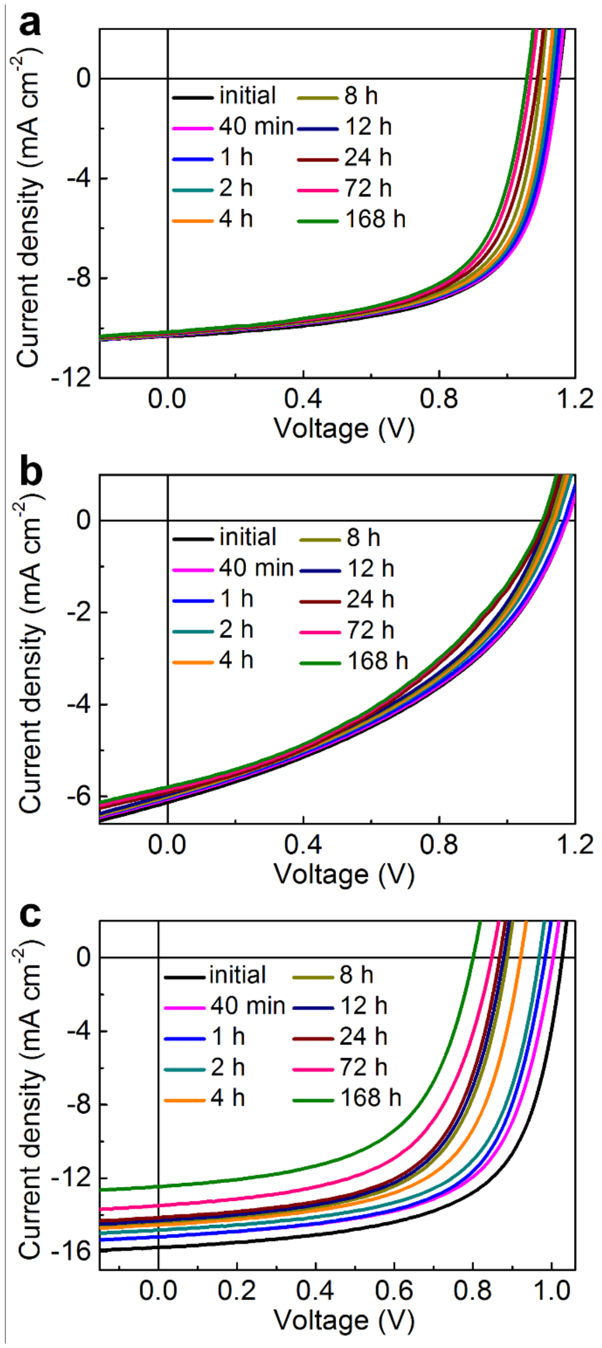

Supplementary Figure 25 *J*−*V* plots of the OSCs based on DR3TBDTC:PBN-11, DR3TBDTT:PBN-11 and PTB7-Th:EH-IDRBR blends after annealing at 180 °C for different time.

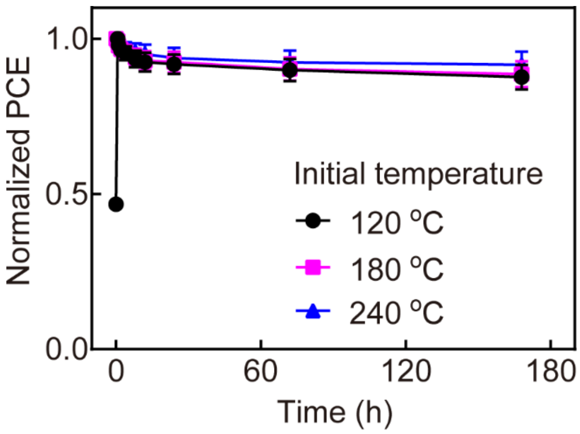

Supplementary Figure 26 The normalized PCE for the OSC devices based on DR3TBDTC:PBN-11 blends with three initial conditions after annealing the active layers at 180 °C for different time. The DR3TBDTC:PBN-11 blends were first annealed at 120, 180 and 240 °C for 20 minutes before the thermal stability test, respectively.

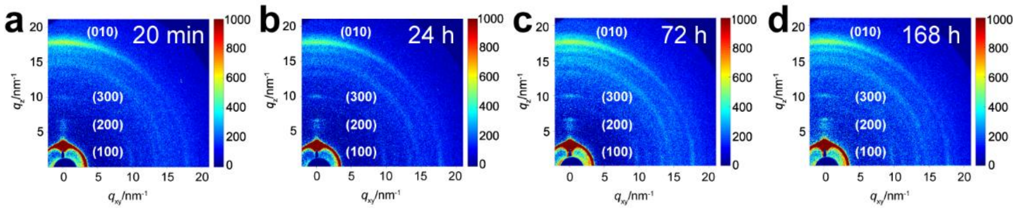

Supplementary Figure 27 GIWAXS patterns of DR3TBDTC:PBN-11 blends annealed at 180 ^{o}C for different time: (a) 20 min, (b) 24 h, (c) 72 h, (d) 168 h.

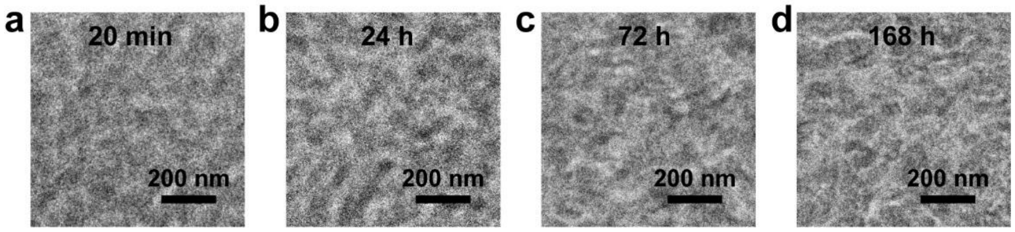

Supplementary Figure 28 TEM images of DR3TBDTC:PBN-11 blends annealed at 180 ºC for different time: (a) 20 min, (b) 24 h, (c) 72 h, (d) 168 h.

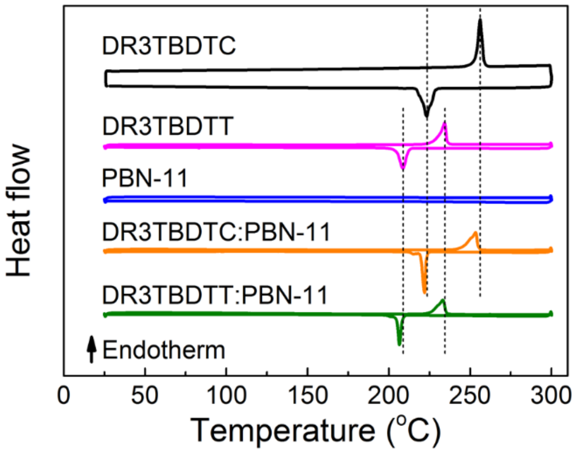

Supplementary Figure 29 DSC second heating and cooling cycles of neat DR3TBDTC, neat DR3TBDTT, neat PBN-11, DR3TBDTC:PBN-11 blend (3:1, w/w) and DR3TBDTT:PBN-11 blend (4:1, w/w) in N_2 atmosphere with a scan rate of 10 °C min^{-1}.

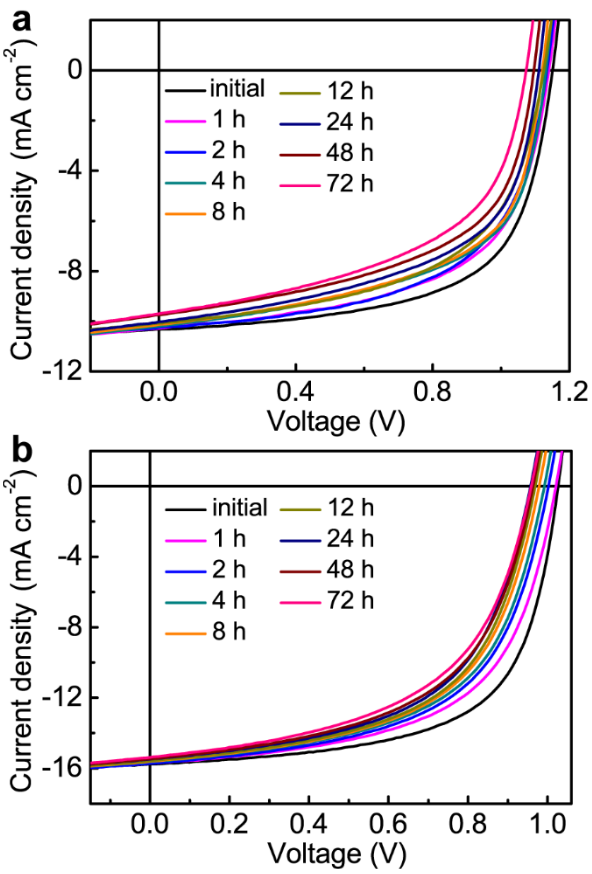

Supplementary Figure 30 *J*−*V* plots of the DR3TBDTC:PBN-11 and PTB7-Th:EH-IDTBR based OSC devices after illumination under 100 mW cm^{-2} AM 1.5G simulated solar light for different time.

Supplementary Tables

Supplementary Table 1 GIWAXS characterization data of the pure DR3TBDTC, DR3TBDTT and PBN-11 films, and the as-cast and thermal annealed DR3TBDTC:PBN-11 and DR3TBDTT:PBN-11 blend films.

**Films**	**Directions**	**100**				**010**			
		**Location (Å^{-1})**	**d-spacing (Å)**	**FWHM (Å^{-1})**	**CL (Å)**	**Location (Å^{-1})**	**d-spacing (Å)**	**FWHM (Å^{-1})**	**CL (Å)**
DR3TBDTC	Out-of-plane	0.309	20.31	0.016	363	1.617	3.89	0.057	100
	In-plane	0.303	20.77	0.011	528	1.619	3.88	0.051	111
DR3TBDTT	Out-of-plane	0.322	19.47	0.015	375				
	In-plane					1.728	3.64	0.070	80
PBN-11	Out-of-plane	0.253	24.81	0.135	42	1.706	3.68	0.162	35
	In-plane	0.258	24.39	0.034	166				
DR3TBDTC:PBN-11 (as-cast)	Out-of-plane	0.313	20.05	0.050	112	1.686	3.73	0.201	28
	In-plane	0.291	21.57	0.051	110				
DR3TBDTT:PBN-11 (as-cast)	Out-of-plane	0.316	19.90	0.035	162				
	In-plane	0.308	20.42	0.029	194	1.714	3.67	0.093	61
DR3TBDTC:PBN-11	Out-of-plane	0.312	20.16	0.039	144	1.674	3.75	0.101	56
	In-plane	0.288	21.82	0.082	69				
		0.308	20.41	0.028	199				
DR3TBDTT:PBN-11	Out-of-plane	0.307	20.46	0.029	194				
	In-plane	0.307	20.47	0.030	212	1.733	3.63	0.092	62

Supplementary Table 2 Photovoltaic performance of the OSCs based on DR3TBDTC:PBN-11 blend films with different weight ratios.

D/A ratio (w/w)	V_{OC} (V)	J_{SC} (mA cm^{-2})	FF (%)	PCE (%)
2/1	1.16 (1.16 ± 0.01)	10.24 (10.10 ± 0.18)	59.8 (58.9 ± 1.1)	7.10 (6.91 ± 0.11)
3/1	1.15 (1.15 ± 0.01)	10.32 (10.31 ± 0.24)	62.7 (61.9 ± 1.3)	7.44 (7.32 ± 0.06)
3/1[a]	1.11 (1.11 ± 0.01)	11.18 (11.16 ± 0.23)	64.6 (64.1 ± 1.4)	8.01 (7.93 ± 0.06)
4/1	1.14 (1.14 ± 0.01)	9.90 (9.81 ± 0.23)	61.7 (60.7 ± 1.5)	6.97 (6.78 ± 0.14)

Data in parentheses are the statistical average and error bars of standard deviation calculated from 16 individual devices and data outside of parentheses are the best devices.

[a] The active area is 2 mm^2.

Supplementary Table 3 Photovoltaic performance of the OSCs based on the DR3TBDTC:PBN-11 blend (3:1, w/w) films annealed at different temperature.

**Temperature (ºC)**	V_{OC} **(V)**	J_{SC} **(mA cm^{-2})**	**FF (%)**	**PCE (%)**
r.t.	1.22 (1.22 ±0.01)	1.57 (1.45 ±0.12)	26.2 (25.6 ±0.7)	0.50 (0.45 ±0.03)
80	1.19 (1.19 ±0.01)	2.98 (2.78 ±0.19)	34.2 (34.1 ±1.2)	1.21 (1.13 ±0.06)
120	1.17 (1.17 ±0.01)	6.85 (6.64 ±0.18)	45.4 (44.9 ±1.2)	3.64 (3.54 ±0.08)
140	1.16 (1.16 ±0.01)	8.99 (8.94 ±0.25)	52.3 (51.6 ±1.0)	5.46 (5.34 ±0.11)
160	1.15 (1.15 ±0.01)	9.91 (9.89 ±0.23)	59.5 (58.4 ±1.1)	6.78 (6.63 ±0.13)
180	1.15 (1.15 ±0.01)	10.32 (10.31 ±0.24)	62.7 (61.9 ±1.3)	7.44 (7.32 ±0.06)
180[a]	1.11 (1.11 ±0.01)	11.18 (11.16 ±0.23)	64.6 (64.1 ±1.4)	8.01 (7.93 ±0.06)
200	1.15 (1.15 ±0.01)	10.11 (9.93 ±0.22)	63.2 (63.0 ±1.4)	7.35 (7.19 ±0.10)
240	1.14 (1.14 ±0.01)	9.89 (9.76 ±0.19)	63.5 (63.3 ±1.4)	7.15 (7.04 ±0.12)

Data in parentheses are the statistical average and error bars of standard deviation calculated from 16 individual devices and data outside of parentheses are the best devices.

[a] The active area is 2 mm^2.

Supplementary Table 4 Photovoltaic performance of the OSCs based on DR3TBDTT:PBN-11 blend films with different weight ratios.

D/A ratio (w/w)	V_{OC} (V)	J_{SC} (mA cm^{-2})	FF (%)	PCE (%)
2:1	1.20 (1.20 ±0.01)	5.65 (5.64 ±0.19)	38.5 (37.5 ±1.0)	2.61 (2.54 ±0.06)
3/1	1.20 (1.20 ±0.01)	6.01 (5.89 ±0.21)	40.0 (39.5 ±1.3)	2.88 (2.78 ±0.06)
4/1	1.18 (1.18 ±0.01)	6.13 (5.99 ±0.25)	40.1 (39.6 ±1.3)	2.90 (2.80 ±0.10)
4/1[a]	1.15 (1.15 ±0.01)	6.21 (6.05 ±0.26)	42.9 (41.7 ±1.4)	3.06 (2.90 ±0.12)
5/1	1.17 (1.17 ±0.01)	5.41 (5.38 ±0.18)	37.6 (36.3 ±1.7)	2.38 (2.28 ±0.10)

Data in parentheses are the statistical average and error bars of standard deviation calculated from 16 individual devices and data outside of parentheses are the best devices.

[a] The active area is 2 mm^2.

Supplementary Table 5 Photovoltaic performance of the OSCs based on DR3TBDTT:PBN-11 blend (4:1, w/w) films annealed at different temperature.

**Temperature (°C)**	V_{OC} **(V)**	J_{SC} **(mA cm^{-2})**	**FF (%)**	**PCE (%)**
r.t.	1.23 (1.23 ±0.01)	2.02 (1.97 ±0.11)	27.1 (26.1 ±0.9)	0.67 (0.63 ±0.03)
80	1.23 (1.23 ±0.01)	2.39 (2.29 ±0.16)	28.4 (27.6 ±1.2)	0.83 (0.78 ±0.04)
120	1.20 (1.20 ±0.01)	3.06 (3.05 ±0.16)	33.1 (31.8 ±1.1)	1.22 (1.16 ±0.04)
140	1.18 (1.18 ±0.01)	4.35 (4.22 ±0.19)	34.6 (34.0 ±1.2)	1.78 (1.69 ±0.07)
160	1.18 (1.18 ±0.01)	5.39 (5.33 ±0.22)	37.3 (36.2 ±1.2)	2.37 (2.27 ±0.07)
180	1.18 (1.18 ±0.01)	6.13 (5.99 ±0.25)	40.1 (39.6 ±1.3)	2.90 (2.80 ±0.10)
180[a]	1.15 (1.15 ±0.01)	6.21 (6.05 ±0.26)	42.9 (41.7 ±1.4)	3.06 (2.90 ±0.12)
200	1.17 (1.17 ±0.01)	6.05 (5.88 ±0.24)	40.1 (39.7 ±1.6)	2.84 (2.72 ±0.10)
240	1.17 (1.17 ±0.01)	5.58 (5.45 ±0.21)	40.5 (39.8 ±1.8)	2.64 (2.52 ±0.11)

Data in parentheses are the statistical average and error bars of standard deviation calculated from 16 individual devices and data outside of parentheses are the best devices.

[a] The active area is 2 mm^2.

Supplementary Table 6 Photovoltaic performance of the OSCs based on DR3TBDTC:PBN-11 blend (3:1, w/w) films annealed at 180 ºC measured in both forward and backward scan directions.

Scan direction	V_{OC} (V)	J_{SC} (mA cm^{-2})	FF (%)	PCE (%)[a]
Forward	1.11	11.18	64.6	8.01
Backward	1.11	11.20	64.3	7.99

[a] The active area is 2 mm^2.

Supplementary Table 7 Photovoltaic parameters of the OSCs based on DR3TBDTC:PBN-11, DR3TBDTT:PBN-11 and DR3TBDTT-BO:PBN-11 blend films.

**Active layers**	**V_{OC} (V)**	**J_{SC} (mA cm^{-2})**	**FF (%)**	**PCE (%)[a]**
DR3TBDTC:PBN-11	1.11 (1.11 ±0.01)	11.18 (11.16 ±0.23)	64.6 (64.1 ±1.4)	8.01 (7.93 ±0.06)
DR3TBDTT:PBN-11	1.15 (1.15 ±0.01)	6.21 (6.05 ±0.26)	42.9 (41.7 ±1.4)	3.06 (2.90 ±0.12)
DR3TBDTT-BO:PBN-11	1.16 (1.15 ±0.01)	6.15 (5.96 ±0.26)	45.6 (45.3 ±1.4)	3.25 (3.10 ±0.10)

Data in parentheses are the statistical average and error bars of standard deviation calculated from 16 individual devices and data outside of parentheses are the best devices.

[a] The active area is 2 mm^2.

Supplementary Table 8 Hole and electron mobilities of DR3TBDTC, DR3TBDTT, PBN-11 film and their blend films.

Films	μ_h (10^{-4} cm^2 V^{-1} s^{-1})	μ_e (10^{-4} cm^2 V^{-1} s^{-1})	μ_h/μ_e
DR3TBDTC	2.15 (1.95 ± 0.15)	/	/
DR3TBDTT	3.37 (3.04 ± 0.20)	/	/
PBN-11	/	6.50 (6.13 ± 0.32)	/
DR3TBDTC:PBN-11	6.97 (6.50 ± 0.36)	2.03 (1.81 ± 0.12)	3.43
DR3TBDTT:PBN-11	1.33 (1.15 ± 0.11)	2.95 (2.72 ± 0.17)	0.45

Data in parentheses are the statistical average and error bars of standard deviation calculated from 16 individual devices and data outside of parentheses are the best optimal results.

Supplementary Table 9 Domain size and domain purity of DR3TBDTC:PBN-11 and DR3TBDTT:PBN-11 their blend films.

Films	Domain size (nm)	Domain purity
DR3TBDTC:PBN-11	30–45, 8–16	0.97
DR3TBDTT:PBN-11	110–130, 40–55	1

Supplementary Table 10 PL quenching efficiency of DR3TBDTC:PBN-11 and DR3TBDTT:PBN-11 their blend films.

**Films**	**PL quenching efficiency**	
	**Excited at 600 nm**	**Excited at 580 nm**
DR3TBDTC:PBN-11	95%	89%
DR3TBDTT:PBN-11	96%	80%

Supplementary Table 11 The melting temperature (T_m), crystallization temperature (T_c), melting enthalpy change (ΔH_m) and crystallization enthalpy change (ΔH_c) of the neat DR3TBDTC, DR3TBDTT and PBN-11, and the DR3TBDTC:PBN-11 (3:1) and DR3TBDTT:PBN-11 (4:1) blends extracted from the second melting and cooling cycles.

**Materials**	**T_m (°C)**	**ΔH_m (J g^{-1})**	**T_c (°C)**	**ΔH_c (J g^{-1})**
DR3TBDTC	256.1	53.5	223.3	52.2
DR3TBDTT	234.3	34.4	208.6	32.0
PBN-11	/	/	/	/
DR3TBDTC:PBN-11	253.3	34.3	221.9	34.2
DR3TBDTT:PBN-11	233.2	26.0	206.5	24.9

Supplementary methods

Materials. Unless noted, all materials were purchased from commercial suppliers and used as received without further purification. PBN-11 was synthesized in our laboratory[1]. 5-bromo-3,3-dioctyl-2,2':5,2"-terthiophene-2-carbaldehyde (compound 6) was prepared according to the reported method[2].

Generals. ^{1}H and ^{13}C NMR spectra were recorded with a Bruker AV-400 spectrometer. MALDITOF-MS was measured with Bruker Daltonics flex Analysis. Elemental analysis was performed with a Bio-Rad elemental analysis system. Thermal gravimetric analysis (TGA) was performed under a nitrogen (N_2) flow with a Perkin-Elmer-TGA 7 system. The temperature of degradation (T_d) corresponded to 5% weight loss. Differential scanning calorimetry (DSC) was performed using a TA DSC Q2000 instrument under nitrogen at the heating and cooling rates of 10 °C min^{-1} in two heating/cooling cycles. The DR3TBDTC:PBN-11 (3:1, w/w) and DR3TBDTT:PBN-11 (4:1, w/w) blend samples for DSC characterization were prepared by drying their active layer solutions from chlorobenzene (CB) on clean glass substrates and collecting the solids from the substrates. Density functional theory (DFT) calculations were performed by Gaussian 09 program. The geometries were optimized at a hybrid B3LYP/6-31G*. For clarity, the alkyl chains were replaced with methyl chains. UV–visible absorption spectra of DR3TBDTC, DR3TBDTT and PBN-11 in solution and in film were measured with a Perkin-Elmer Lambda 35 UV−vis spectrometer. The solution samples were prepared using CB solvent with a concentration of 1×10^{-5} M. The film samples were fabricated by spin-coating the warm CB solutions onto quartz

substrates and then annealed at 180 ℃ for 20 min. Photoluminescence (PL) spectra were measured with a Horiba Jobin-Yvon FL3C-111 fluorescence spectrophotometer. The pure and blend films were prepared from their warm CB solution and annealed at 180 ℃ for 20 min. The exciting wavelengths are 600 nm for PBN-11 and 580 nm for DR3TBDTC or DR3TBDTT. Cyclic voltammetry (CV) was performed on a CHI660a electrochemical workstation using *n*-Bu_4NPF_6 (0.1 M) in acetonitrile as electrolyte solution and ferrocene as an internal reference at a scan rate of 20 mV s^{-1}. The CV cell consisted of a glassy carbon electrode, a Pt wire counter electrode, and a standard calomel reference electrode. The redox potentials were calibrated with ferrocene as an internal standard. The highest occupied molecular orbital (HOMO) and the lowest unoccupied molecular orbital (LUMO) energy levels were estimated by the equations: $E_{HOMO}/_{LUMO} = -(4.80 + E_{onset}^{ox}/E_{onset}^{red})$ eV. The transmission electron microscopy (TEM) measurement was performed on a TECNAI G2 transmission electron microscope (FEI Co., Netherlands) operating at 200 kV. The atomic force microscopy (AFM) characterization was performed on a SPA 300HV with a SPI 3800N controller (Seiko Instruments, Inc., Japan) in tapping mode. A silicon micro cantilever (spring constant 2 N m^{-1} and resonance frequency ca. 300 kHz, Olympus Co., Japan) with an etched conical tip was used for the scan. The thickness of films was measured with a XP-plus Stylus Profilometer.

Two-dimensional grazing incidence wide-angle X-ray scattering (2D-GIWAXS) characterization. GIWAXS measurements were performed at beamline 7.3.3 at the Advanced Light Source (ALS) or BL1W1A beamline at Beijing Synchrotron Radiation

Facility (BSRF)[3]. Samples were prepared on Si substrates. The samples for the pure films were fabricated using their CB solutions and annealed at 180 °C for 20 min. Both the CB solutions and Si substrates were preheated at 80 °C. The samples for the blend films were fabricated using identical blend solutions as those used in devices. Both the as-cast and annealed blend films (180 °C for 20 min) were prepared. For the beamline 7.3.3 at ALS, the 10 keV X-ray beam was incident at a grazing angle of 0.12°–0.16°, selected to maximize the scattering intensity from the samples. The scattered X-rays were detected using a Dectris Pilatus 2M photon counting detector. For BL1W1A beamline at BSRF, the incidence angle is 0.16° and the scattered X-rays were detected using a Mar 345 detector.

Resonant soft X-ray scattering (R-SoXS) characterization. R-SoXS transmission measurements were performed at beamline 11.0.1.2 at the Advanced Light Source (ALS)[4,5]. Samples for R-SoXS measurements were prepared on a PSS modified Si substrate under the same conditions as those used for device fabrication, and then transferred by floating in water to a 1.5 mm × 1.5 mm, 100 nm thick Si_3N_4 membrane supported by a 5 mm × 5 mm, 200 μm thick Si frame (Norcada Inc.). 2D scattering patterns were collected on an in-vacuum CCD camera (Princeton Instrument PI-MTE). The sample detector distance was calibrated from diffraction peaks of a triblock copolymer poly(isoprene-b-styrene-b-2-vinyl pyridine), which has a known spacing of 391 Å. The beam size at the sample is approximately 100 μm by 200 μm.

Synthesis of compound 2. Under the protection of argon, a mixture of 9*H*-carbazole (**1**) (7.00 g, 41.86 mmol), sodium hydride (2.31 g, 96.25 mmol) and anhydrous *N,N*-

dimethylformamide (160 mL) were stirred at room temperature for 0.5 h. Then, 3-(bromomethyl)heptane (9.70 g, 50.23 mmol) was added to the mixture, followed by stirred at room temperature for 30 h. After workup, the mixture was poured to water and extracted with dichloromethane for three times. The organic phase was dried over anhydrous Na_2SO_4 and the solvent was then removed by rotary evaporation. The crude product was purified by silica gel chromatography with petroleum ether to give **2** (8.10 g, yielded 69%) as a colourless oil. ^{1}H NMR (400 MHz, $CDCl_3$): δ (ppm) 8.09-8.07 (d, J = 7.8 Hz, 2H), 7.45–7.41 (t, J = 7.6 Hz, 2H), 7.38–7.36 (d, J = 8.1 Hz, 2H), 7.22–7.19 (t, J = 7.4 Hz, 2H), 4.18–4.08 (m, 2H), 2.07–2.04 (m, 1H), 1.42–1.22 (m, 8H), 0.91–0.83 (m, 6H). ^{13}C NMR (100 MHz, $CDCl_3$): δ (ppm) 140.99, 125.58, 122.85, 120.32, 118.72, 109.00, 47.45, 39.45, 31.08, 28.89, 24.47, 23.12, 14.09, 10.97.

Synthesis of compound 3. To the solution of 9-(2-ethylhexyl)-9*H*-carbazole (**2**) (7.84 g, 28.06 mmol) in chloroform (150 mL), *N*-bromosuccinimide (4.99 g, 28.06 mmol) was added at 0 °C in dark. The mixture was stirred at 60 °C for 12 h. Then, the reaction mixture was poured into water and extracted with dichloromethane. The organic phase was washed with water for three times, dried over anhydrous Na_2SO_4, filtered and concentrated. The crude product was purified by silica gel chromatography with 5:1 (v/v) petroleum ether–dichloromethane to give **3** (10.05 g, yielded 100%) as a yellow oil. ^{1}H NMR (400 MHz, $CDCl_3$): δ (ppm) 8.17 (s, 1H), 8.03–8.01 (d, J = 7.8 Hz, 1H), 7.51–7.44 (m, 2H), 7.37–7.35 (d, J = 8.2 Hz, 1H), 7.24–7.20 (t, J = 8.0 Hz, 2H), 4.13–4.05 (m, 2H), 2.05–1.98 (m, 1H), 1.38–1.21 (m, 8H), 0.91–0.83 (m, 6H). ^{13}C NMR (100 MHz, $CDCl_3$): δ (ppm) 141.18, 139.55, 128.18, 126.29, 124.52, 123.01, 121.78,

120.48, 119.16, 111.49, 110.40, 109.22, 47.52, 39.36, 31.00, 28.76, 24.39, 23.04, 14.03, 10.90.

Synthesis of compound 4. *n*-Butyllithium (*n*-BuLi) (10.87 mL, 2.5 mol L^{-1}) was added dropwise to the solution of 3-bromo-9-(2-ethylhexyl)-9*H*-carbazole (**3**) (9.53 g, 26.60 mmol) in anhydrous THF (120 mL) at −78 °C under argon atmosphere. After stirred at −78 °C for 2 h, the mixture was added benzo[1,2-*b*:4,5-*b'*]dithiophene-4,8-dione (2.55 g, 11.58 mmol), followed by stirred at 50 °C for 2 h. Then the reaction mixture was cooled to ambient temperature to added in a solution of $SnCl_2{\cdot}2H_2O$ (16.15 g, 71.57 mmol) in 10% HCl (73 mL). The reaction mixture was stirred for another 2 h and then poured into ice water. The mixture was extracted twice with dichloromethane. The organic phase was washed with water for three times, dried over anhydrous Na_2SO_4, filtered and concentrated. The crude product was purified by silica gel chromatography with 6:1 (v/v) petroleum ether–dichloromethane to give **4** (3.45 g, yielded 40%) as a yellow solid. ^{1}H NMR (400 MHz, $CDCl_3$): δ (ppm) 8.47 (s, 2H), 8.13–8.12 (d, J = 7.7 Hz, 2H), 7.84–7.82 (d, J = 8.4 Hz, 2H), 7.59–7.57 (d, J = 8.4 Hz, 2H), 7.52–7.42 (m, 6H), 7.41–7.39 (d, J = 5.6 Hz, 2H), 7.27–7.23 (t, J = 7.2 Hz, 2H), 4.31–4.19 (m, 4H), 2.20–2.14 (m, 2H), 1.50–1.29 (m, 16H), 1.01–0.87 (m, 12H). ^{13}C NMR (100 MHz, $CDCl_3$): δ (ppm) 141.48, 140.69, 138.84, 136.65, 131.04, 130.06, 127.08, 127.03, 125.91, 123.42, 123.18, 122.87, 121.31, 120.48, 119.02, 109.22, 109.19, 47.70, 39.58, 31.11, 28.89, 24.54, 23.10, 14.09, 11.00.

Synthesis of compound 5. *n*-Butyllithium (*n*-BuLi) (3.77 mL, 2.5 mol L^{-1}) was added dropwise to the solution of 4,8-bis(9-(2-ethylhexyl)-9*H*-carbazol-3-yl)benzo[1,2-*b*:4,5-

b']dithiophene (**4**) (2.81 g, 3.77 mmol) in anhydrous THF (100 mL) at −78 °C under argon atmosphere. After stirred at −78 °C for 40 min, the reaction mixture was stirred for another 1 h at room temperature. Then chlorotrimethylstannane was added to the mixture at −78 °C, followed by stirred at room temperature for 12 h. The reaction mixture was poured into a solution of KF and extracted with dichloromethane. The organic phase was washed with water for three times, dried over anhydrous Na_2SO_4, filtered and concentrated. The crude product was purified by recrystallization in dichloromethane/*n*-hexane to give **5** (3.23 g, yielded 80%) as a yellow solid. ^{1}H NMR (400 MHz, C_6D_6): δ (ppm) 8.80 (s, 2H), 8.21–8.19 (d, *J* = 8.4 Hz, 2H), 8.03–8.01 (d, *J* = 7.4 Hz, 2H), 7.00 (s, 2H), 7.45–7.43 (m, 4H), 7.33–7.31 (d, *J* = 8.4 Hz, 2H), 7.22–7.18 (t, *J* = 7.5 Hz, 2H), 3.87–3.77 (m, 4H), 2.03–1.88 (m, 2H), 1.35–1.00 (m, 16H), 0.85–0.81 (t, *J* = 6.4 Hz, 6H), 0.73–0.69 (t, *J* = 7.2 Hz, 6H), 0.15 (s, 18H). ^{13}C NMR (100 MHz, C_6D_6): δ (ppm) 144.21, 141.97, 141.80, 141.74, 140.95, 138.51, 131.66, 131.61, 130.39, 125.99, 123.93, 123.53, 121.97, 121.07, 119.42, 109.63, 109.20, 47.39, 39.39, 31.11, 28.78, 24.51, 23.21, 14.09, 10.80, -8.92.

Synthesis of compound 7. A mixture of 3,3'-(2,6-bis(trimethylstannyl)benzo[1,2-*b*:4,5-*b'*]dithiophene-4,8-diyl)bis(9-(2-ethylhexyl)-9*H*-carbazole) (**5**) (1.40 g, 1.31 mmol), compound (**6**) (1.74 g, 3.00 mmol), $Pd(PPh_3)_4$ (0.39 g, 0.34 mmol) and anhydrous toluene (130 mL) were stirred with argon at 110 °C for 24 h. After removal of solvent, the crude product was purified by silica gel chromatography with 1:2 (v/v) petroleum ether–chloroform to give **7** (1.45 g, yielded 64%) as a red solid. ^{1}H NMR (400 MHz, $CDCl_3$): δ (ppm) 9.80 (s, 2H), 8.48 (s, 2H), 8.19–8.17 (d, *J* = 7.7 Hz, 2H),

7.85–7.83 (d, *J* = 8.4 Hz, 2H), 7.64–7.62 (d, *J* = 8.4 Hz, 2H), 7.56 (s, 2H), 7.53–7.48 (m, 4H), 7.45 (s, 2H), 7.30–7.26 (d, *J* = 7.3 Hz, 2H), 7.19–7.18 (d, *J* = 3.8 Hz, 2H), 7.05 (s, 4H), 4.34–4.24 (m, 4H), 2.82–2.74 (t, *J* = 8.0 Hz, 4H), 2.74–2.65 (t, *J* = 8.0 Hz, 4H), 2.24–2.12 (m, 2H), 1.72–1.13 (m, 64H), 1.08–0.99 (t, *J* = 7.6 Hz, 6H), 0.97–0.88 (t, *J* = 7.6 Hz, 6H), 0.88–0.75 (m, 12H). ^{13}C NMR (100 MHz, $CDCl_3$): δ (ppm) 182.47, 141.56, 141.09, 140.98, 140.81, 140.33, 140.23, 138.97, 138.74, 138.00, 137.57, 137.20, 136.03, 134.57, 130.59, 129.95, 129.58, 128.18, 127.82, 126.93, 126.16, 126.07, 123.33, 122.82, 121.18, 120.60, 119.23, 119.12, 109.48, 109.27, 47.75, 39.64, 31.83, 31.11, 30.42, 30.26, 29.62, 29.58, 29.44, 29.37, 29.22, 29.19, 28.88, 24.56, 23.16, 22.63, 14.11, 14.07, 11.01, –0.01.

Synthesis of DR3TBDTC. Compound (**7**) (0.20 g, 0.11 mmol) and 3-ethylrhodanine (0.33 g, 2.07 mmol) were dissolved in anhydrous $CHCl_3$ (50 mL), and then three drops of piperidine were added to the mixture. After refluxed for 10 h, the reaction mixture was extracted by water and chloroform for three times. The organic phase was dried over anhydrous Na_2SO_4 and the solvent was then removed by rotary evaporation. The crude product was purified by silica gel chromatography with 1:4 (v/v) petroleum ether–chloroform and recrystallization in *n*-hexane/chloroform to give DR3TBDTC (0.20 g, yielded 86%) as a brown solid. ^{1}H NMR (400 MHz, $CDCl_3$): δ (ppm) 8.49 (s, 2H), 8.19–8.18 (d, *J* = 7.7 Hz, 2H), 7.85–7.83 (d, *J* = 8.3 Hz, 2H), 7.76 (s, 2H), 7.65–7.63 (d, *J* = 8.4 Hz, 2H), 7.55–7.48 (m, 4H), 7.45 (s, 2H), 7.31–7.29 (d, *J* = 7.1 Hz, 2H), 7.20 (s, 2H), 7.17–7.16 (d, *J* = 3.8 Hz, 2H), 7.06 (s, 4H), 4.35–4.25 (m, 4H), 4.21–4.15 (q, *J* = 6.8 Hz, *J* = 7.2 Hz, 4H), 2.80–2.76 (t, *J* = 8.0 Hz, 4H), 2.73–2.70 (t, *J* = 8.0 Hz,

4H), 2.24–2.18 (m, 2H), 1.68–1.58 (m, 8H), 1.56–1.14 (m, 62H), 1.07–0.98 (t, *J* = 7.2 Hz, 6H), 0.97–0.89 (t, *J* = 7.2 Hz, 6H), 0.88–0.79 (m, 12H). ^{13}C NMR (100 MHz, $CDCl_3$): δ (ppm) 192.02, 167.31, 141.56, 141.08, 141.00, 140.80, 139.46, 138.70, 137.67, 137.57, 137.27, 137.20, 135.96, 135.20, 134.61, 130.57, 130.04, 129.58, 128.17, 127.29, 126.93, 126.91, 126.18, 126.06, 124.89, 123.33, 122.82, 121.19, 120.61, 120.56, 119.22, 119.11, 109.47, 109.26, 47.74, 39.90, 39.64, 31.84, 31.83, 31.11, 30.38, 30.23, 29.70, 29.64, 29.58, 29.48, 29.39, 29.37, 29.21, 29.20, 28.88, 24.56, 23.16, 22.63, 14.11, 14.07, 12.27, 11.02, -0.01.MS (MALDITOF) *m/z*: Calculated for $C_{118}H_{138}N_4O_2S_{12}$ $[M]^+$,2026.7; found 2026.7. Elemental analyses calculated (%) for $C_{118}H_{138}N_4O_2S_{12}$ (DR3TBDTC): C, 69.84; H, 6.85; N, 2.76. Found: C, 69.80; H, 6.82; N, 2.77.

Supplementary References

1. Long, X. *et al.* Double B←N bridged bipyridine-containing polymer acceptors with enhanced electron mobility for all-polymer solar cells. *Mater. Chem. Front.* **3**, 70–77 (2019).
2. Zhou, J. *et al.* A planar small molecule with dithienosilole core for high efficiency solution-processed organic photovoltaic cells. *Chem. Mater.* **23**, 4666–4668 (2011).
3. Hexemer, A. *et al.* A SAXS/WAXS/GISAXS beamline with multilayer monochromator. *J. Phys: Conf. Ser.* **247**, 012007 (2010).
4. Gann, E. *et al.* Soft x-ray scattering facility at the Advanced Light Source with real-time data processing and analysis. *Rev. Sci. Instrum.* **83**, 045110 (2012).
5. Wu, Y. *et al.* Morphology analysis of organic solar cells with synchrotron radiation based resonant soft x-ray scattering. *Prog. Chem.* **29**, 93–101 (2017).
