# Supplementary material for: Efficient and thermally stable organic solar cells based on small molecule donor and polymer acceptor
Source: Nat Commun. 2019 Jul 22;10:3271. doi: 10.1038/s41467-019-10984-6 (PMC6646397; doi:10.1038/s41467-019-10984-6)
Supplement: Supplementary file 3 — Solar Cells Reporting Summary [file 41467_2019_10984_MOESM3_ESM.pdf]

nature research

Corresponding author(s): Jun Liu

Solar Cells Reporting Summary

Nature Research wishes to improve the reproducibility of the work that we publish. This form is intended for publication with all accepted papers reporting the characterization of photovoltaic devices and provides structure for consistency and transparency in reporting. Some list items might not apply to an individual manuscript, but all fields must be completed for clarity.

For further information on Nature Research policies, including our data availability policy, see Authors & Referees.

Experimental design

Please check: are the following details reported in the manuscript?

1. Dimensions

Item	Yes	No	Details
Area of the tested solar cells	☒	☐	Described in Methods, section "OSC devices fabrication and measurement"
Method used to determine the device area	☒	☐	Described in Methods, section "OSC devices fabrication and measurement".

2. Current-voltage characterization

Item	Yes	No	Details
Current density-voltage (J-V) plots in both forward and backward direction	☒	☐	Provided in Supplementary Fig. 17.
Voltage scan conditions<br>*For instance: scan direction, speed, dwell times*	☒	☐	Described in Methods, section "OSC devices fabrication and measurement".
Test environment<br>*For instance: characterization temperature, in air or in glove box*	☒	☐	Described in Methods, section "OSC devices fabrication and measurement".
Protocol for preconditioning of the device before its characterization	☐	☒	No preconditioning of the device before its characterization.
Stability of the J-V characteristic<br>*Verified with time evolution of the maximum power point or with the photocurrent at maximum power point; see ref. 7 for details.*	☒	☐	Provided in Figure 6.

3. Hysteresis or any other unusual behaviour

Item	Yes	No	Details
Description of the unusual behaviour observed during the characterization	☐	☒	No unusual behavior was observed.
Related experimental data	☐	☒	No unusual behavior was observed.

4. Efficiency

Item	Yes	No	Details
External quantum efficiency (EQE) or incident photons to current efficiency (IPCE)	☒	☐	Provided in Figure 3.
A comparison between the integrated response under the standard reference spectrum and the response measure under the simulator	☒	☐	Provided in Table 2.
For tandem solar cells, the bias illumination and bias voltage used for each subcell	☐	☒	No tandem solar cells are involved in this manuscript.

5. Calibration

Item	Yes	No	Details
Light source and reference cell or sensor used for the characterization	☒	☐	Described in Methods, section "OSC devices fabrication and measurement".
Confirmation that the reference cell was calibrated and certified	☒	☐	Described in Methods, section "OSC devices fabrication and measurement".

November 2017

Calculation of spectral mismatch between the reference cell and the devices under test	☐ Yes ☒ No	Spectral mismatch was not calculated. By calculating the Jsc from EQE based on the solar simulator spectral for the standard silicon cell and our OSC devices, the mismatch factor was close to unity.

6. Mask/aperture

Size of the mask/aperture used during testing	☒ Yes ☐ No	Described in Methods, section "OSC devices fabrication and measurement".
Variation of the measured short-circuit current density with the mask/aperture area	☒ Yes ☐ No	Provided in Supplementary Table 2.

7. Performance certification

Identity of the independent certification laboratory that confirmed the photovoltaic performance	☐ Yes ☒ No	Limited by the encapsulation, we have not certified our efficiency values.
A copy of any certificate(s) *Provide in Supplementary Information*	☐ Yes ☒ No	No certification.

8. Statistics

Number of solar cells tested	☒ Yes ☐ No	Provided in Table 2.
Statistical analysis of the device performance	☒ Yes ☐ No	Provided in Table 2.

9. Long-term stability analysis

Type of analysis, bias conditions and environmental conditions *For instance: illumination type, temperature, atmosphere humidity, encapsulation method, preconditioning temperature*	☒ Yes ☐ No	Described in Methods, section "Thermal and light stability tests".
